# ENGAGING STRATEGIES IN CLASSES, COMMUNITIES, AND RESEARCH

**DOI:** 10.1093/geroni/igad104.1943

**Published:** 2023-12-21

**Authors:** Betsy Kemeny

**Affiliations:** Slippery Rock University, Grove City, Pennsylvania, United States

## Abstract

Educators can use community engaged learning (CEL), also known as service learning, to empower all ages, bridging both college students and older adults needs. With the wrong approach, service just attached to a course may reinforce negative stereotypes. To assure best practice, educators benefit from CEL competencies to promote positive learning and avoid reinforcing ageism. Competencies, based on a community-engaged taxonomy, include: Reciprocal partnerships, Diversity of interactions and dialogue,​ Community activities, Civic competencies, Critical reflection, and Assessment (Rathlef, 2022; Kecskes, 2015). A complete explanation of each competency will be explained and illustrated. Using the competencies as a framework, two specific programs with college students and older adults were evaluated using both formative and summative measures. One program, a virtual intergenerational interaction, lasted seven weeks. The second program, an in-person program at the University’s equestrian and nature center, spanned five weeks. Pre and post comparisons of civic learning outcomes using CLEO, written reflections, focus group discussion, and partner perspectives were analyzed. Older adults reported some improvement in social interaction and companionship with some expansion in social networks. Students significantly improved civic knowledge outcomes. The students’ written reflections revealed a self-reported change in attitude toward older adults. Students also expressed a shift in their perceptions of future work in a health setting with older adults. Community-engaged learning can empower all ages, but an educator’s attention to the competencies supporting best practice can make a significant difference to the outcome of these efforts.

